# Blockage of mechanosensitive Piezo1 channel alleviates the severity of experimental malaria-associated acute lung injury

**DOI:** 10.1186/s13071-024-06144-5

**Published:** 2024-02-01

**Authors:** Min Zhang, Qian Ru Wang, Xinpeng Hou, Qi Wang, Xiaoyan Yang, Tingting Zhou, Xiaobo Liu, Lirong Wu, Jie Wang, Xiaobao Jin, Zhenlong Liu, Bo Huang

**Affiliations:** 1 Guangdong Provincial Key Laboratory of Pharmaceutical Bioactive Substances, Guangdong Pharmaceutical University, Guangzhou, 510006 People’s Republic of China; 2 Guangzhou Chest Hospital, Guangzhou, 510095 People’s Republic of China; 3 Department of Laboratory Medicine, Central Hospital of Panyu District, Guangzhou, 511400 People’s Republic of China; 4 School of Basic Medical Science, Guangdong Pharmaceutical University, Guangzhou, 510006 People’s Republic of China; 5 Division of Experimental Medicine, Department of Medicine, McGill University, Montreal, QC Canada

**Keywords:** Malaria, Acute lung injury, Piezo1, Macrophage polarization, Ferroptosis

## Abstract

**Background:**

Malaria-associated acute lung injury (MA-ALI) is a well-recognized clinical complication of severe, complicated malaria that is partly driven by sequestrations of infected red blood cells (iRBCs) on lung postcapillary induced impaired blood flow. In earlier studies the mechanosensitive Piezo1 channel emerged as a regulator of mechanical stimuli, but the function and underlying mechanism of Piezo1 impacting MA-ALI severity via sensing the impaired pulmonary blood flow are still not fully elucidated. Thus, the present study aimed to explore the role of Piezo1 in the severity of murine MA-ALI.

**Methods:**

Here, we utilized a widely accepted murine model of MA-ALI using C57BL/6 mice with *Plasmodium berghei* ANKA infection and then added a Piezo1 inhibitor (GsMTx4) to the model. The iRBC-stimulated Raw264.7 macrophages in vitro were also targeted with GsMTx4 to further explore the potential mechanism.

**Results:**

Our data showed an elevation in the expression of Piezo1 and number of Piezo1^+^-CD68^+^ macrophages in lung tissues of the experimental MA-ALI mice. Compared to the infected control mice, the blockage of Piezo1 with GsMTx4 dramatically improved the survival rate but decreased body weight loss, peripheral blood parasitemia/lung parasite burden, experimental cerebral malaria incidence, total protein concentrations in bronchoalveolar lavage fluid, lung wet/dry weight ratio, vascular leakage, pathological damage, apoptosis and number of CD68^+^ and CD86^+^ macrophages in lung tissues. This was accompanied by a dramatic increase in the number of CD206^+^ macrophages (M2-like subtype), upregulation of anti-inflammatory cytokines (e.g. IL-4 and IL-10) and downregulation of pro-inflammatory cytokines (e.g. TNF-α and IL-1β). In addition, GsMTx4 treatment remarkably decreased pulmonary intracellular iron accumulation, protein level of 4-HNE (an activator of ferroptosis) and the number of CD68^+^-Piezo1^+^ and CD68^+^-4-HNE^+^ macrophages but significantly increased protein levels of GPX4 (an inhibitor of ferroptosis) in experimental MA-ALI mice. Similarly, in vitro study showed that the administration of GsMTx4 led to a remarkable elevation in the mRNA levels of CD206, IL-4, IL-10 and GPX-4 but to a substantial decline in CD86, TNF-α, IL-1β and 4-HNE in the iRBC-stimulated Raw264.7 cells.

**Conclusions:**

Our findings indicated that blockage of Piezo1 with GsMTx4 alleviated the severity of experimental MA-ALI in mice partly by triggering pulmonary macrophage M2 polarization and subsequent anti-inflammatory responses but inhibited apoptosis and ferroptosis in lung tissue. Our data suggested that targeting Piezo1 in macrophages could be a promising therapeutic strategy for treating MA-ALI.

**Graphical Abstract:**

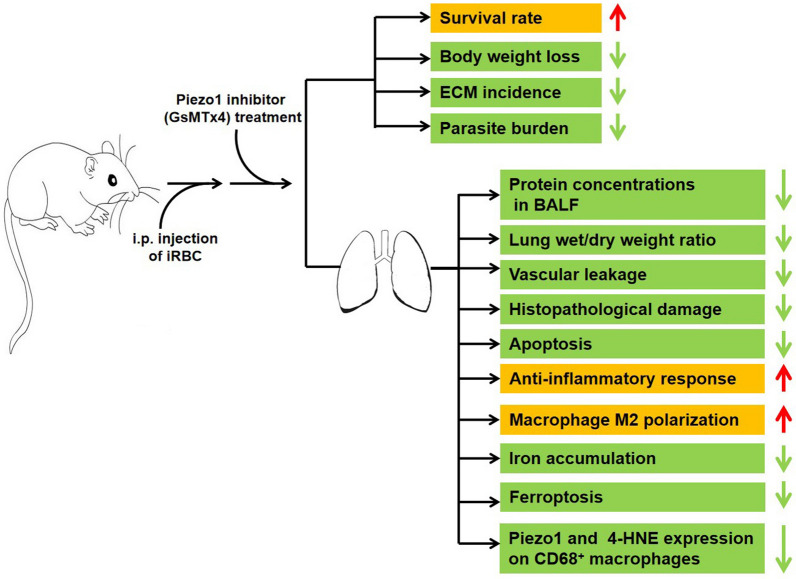

**Supplementary Information:**

The online version contains supplementary material available at 10.1186/s13071-024-06144-5.

## Background

Malaria, which is caused by bites of female *Anopheles* mosquitoes carrying *Plasmodium* spp., is highly endemic in sub-Saharan Africa, Southeast Asia and South America and results in enormous economic losses in endemic areas [1]. Even after adequate therapeutic management (e.g. artemisinin-based combination therapy) and preventive strategies (e.g. indoor residual spraying and long-lasting insecticide-treated nets), malaria remains one of the most severe public health problems worldwide, affecting ~ 241 million individuals, and results in the deaths of ~ 627,000 people annually [1]. To achieve its life cycle, *Plasmodium* parasites successively invade and replicate in the human host’s liver cells and red blood cells (RBCs) and subsequently trigger liver damage and anemia. Malaria infections affect other organs (e.g. brain, kidney and lung) and lead to multiple organ-specific dysfunctions [e.g. cerebral malaria (CM), acute kidney injury and acute lung injury (ALI)] by inducing uncontrolled or excessive inflammatory responses. Malaria associated-ALI (MA-ALI), and its more severe form (malaria associated-acute respiratory distress syndrome, MA-ARDS), is a well-recognized and life-threatening complication of severe *Plasmodium* spp. infection that is usually characterized by excessive pulmonary inflammation, leukocyte recruitment, pulmonary edema and alveolar epithelial damage, ultimately resulting in respiratory failure and high morbidity and mortality rates [2]. Many clinical studies have demonstrated that about 20–30% of severe *Plasmodium falciparum* malaria patients experience MA-ALI, and 50–90% die, even after anti-malarial treatment has remarkably reduced parasitemia levels [3]. Accumulated studies have shown that sequestration of iRBCs and leukocytes on lung postcapillary venules, followed by impaired blood flow, endothelial cell dysfunction, macrophage recruitment and excessive pro-inflammatory responses, are key factors in the severity of MA-ALI [4, 5], yet its underlying pathogenetic mechanism is not well elucidated.

Piezo1, a newly identified non-selective mechanosensitive ion channel, is widely distributed in a variety of cells and organs (e.g. lungs, skin and kidneys) that rapidly sense multi-mechanical stimuli (e.g. changes in blood flow and blood pressure) and ultimately is involved in pathophysiological processes of numerous lung diseases [6]. It was reported that pulmonary mechanical status changes (e.g. impaired blood flow) were primarily triggered by sequestrations of iRBCs on lung postcapillary venules in both malaria patients with ALI and a murine model of MA-ALI [7], indicating that Piezo1 may be involved in the pathogenic process of MA-ALI via sensing impaired pulmonary blood flow. Previous works showed that human RBCs from African individuals carrying gain-of-function PIEZO1 allele (E756del) were dehydrated and could attenuate intracellular growth of *P. falciparum* strain in vitro, indicating that Piezo1 in RBCs had significant resistance against severe malaria [8, 9]. Similarly, an in vitro study showed that human RBC infection by *P. falciparum* strain invasion was efficiently suppressed by the pharmacological activation of Piezo1 with Yoda1 via RBC dehydration [10]. A growing body of evidence has shown that Piezo1 transduced mechanical force to myeloid cells (e.g. leukocytes, monocytes and macrophages) and served as a vital regulator of innate immune responses with implications for tumor immunity and pathogen infections [11, 12]. Of note, although the link between Piezo1 in RBCs and inhibition of *Plasmodium* parasite growth has been extensively investigated so far, no data on the outcome of Piezo1 regarding the severity of MA-ALI via meditating host immune cell (e.g. macrophage) function and immune responses have yet been described.

Pulmonary macrophages, which comprise alveolar and interstitial macrophages, are one of the most abundant and key innate immune cells in lung tissue and subsequently play a key role in the first-line defense against pathogens, tissue repair and immune regulation by phagocytosis and appropriate inflammatory responses [13]. Alternatively, pulmonary macrophage dysfunction can exacerbate lung injury in a variety of diseases, including MA-ALI [14]. Upon multiple stimuli, macrophages are usually polarized into two distinct subsets: (i) M1-like macrophages that produce massive pro-inflammatory cytokines and are closely linked to host defense against pathogens; (ii) M2-like macrophages that alternatively contribute to tissue repair by triggering an anti-inflammatory response. Accumulating evidence has demonstrated that macrophages play a conflicting role (e.g. as a protective or pathological factor) in response to malaria infections, including MA-ALI [15]. M1-like macrophages were prominent in lung tissues from severe *P. falciparum* malaria patients with pulmonary edema and served as a key factor in triggering lung pathology [5]. Alternatively, IL-33-mediated protection against experimental CM (ECM) was linked to induction of M2-like macrophages [16]. Conversely, pre-existing infection of *Trichinella spiralis* suppressed *Plasmodium berghei* parasitemia but aggravated malaria-induced liver pathology partly by stimulating M2 macrophages [17]. Higher expression of CD11b and HIF-1α in pulmonary macrophages plays a central role in mediating alveolar-endothelial barrier disruption, which ultimately leads to lung injury in malaria patients with ALI [18]; in other work, alveolar macrophages were conversely proposed as a protective agent in response to malaria infection, since the anti-CD11b treatment diminished monocyte homing and decreased parasite elimination in the lung tissues [19]. Based on these findings, the trend and severity of MA-ALI may be determined by the imbalance of pulmonary M1/M2 macrophages, and it is suggested that the control of macrophage M1/M2 polarization may be expected to improve the severity of MA-ALI. Currently, several studies have demonstrated that Piezo1 is highly expressed in macrophages and involved in modulating immune functional response of macrophages, including M1/M2 polarization [20, 21]. A further report indicates the role of perivascular macrophages as regulators of blood flow following tissue damage [22]. Given that impaired blood flow induced by sequestration of iRBCs on lung postcapillary venules was commonly observed in lung tissue of malaria patients with ALI and experimental MA-ALI mice, these findings raise an intriguing possibility that Piezo1 in macrophages could mediate the severity of MA-ALI by sensing impaired pulmonary blood flow. So far, its mechanism is unknown.

Because of limitations in accessing lung tissues from malaria patients with ALI, a murine model of C57BL/6 mice with *P. berghei* ANKA strain infection has usually been adopted to explore the pathogenesis of MA-ALI; this model exhibited various similarities of syndromes in malaria patients with ALI, including iRBC sequestration on lung postcapillary venules, pulmonary edema and hemorrhage [23]. Herein, this study administered a Piezo1 inhibitor (GsMTx4) to experimental MA-ALI mice by using C57BL/6 mice with *P. berghei* ANKA infection to investigate the function of Piezo1 in the pathogenesis of MA-ALI. iRBC-stimulated mouse Raw264.7 macrophage cells were also targeted with GsMTx4 to further explore the potential mechanism. Using in vivo and in vitro assays, our findings demonstrated that blockage of Piezo1 with GsMTx4 dramatically triggered pulmonary macrophage M2 polarization and subsequent anti-inflammatory responses, but suppressed apoptosis and ferroptosis, which in turn alleviated the severity of MA-ALI. Our findings could update the current understanding of the link between Piezo1 and severity of MA-ALI, and we propose that blocking Piezo1 in macrophages may be a valuable therapeutic approach for treating MA-ALI.

## Methods

### Mice and ethics statement

Female C57BL/6 mice (6 weeks old, weighing ~ 20 g) were purchased from Guangdong Medical Experimental Animal Center and bred in a specific pathogen-free (SPF) facility with free access to food/water as well as a standard 12-h light/dark cycles in the Experimental Animal Center of Guangdong Pharmaceutical University, China. *Plasmodium berghei* ANKA-iRBCs (kindly donated by Prof. Jianping Song, Guangzhou University of Chinese Medicine, China) were stored in our laboratory's − 80 °C freezer and kept by regular passage in donor mice with intraperitoneal (i.p.) injection. All animal experiments were conducted following the National Guidelines for the Care and Use of Animals and approved by the Ethics Committee of Guangdong Pharmaceutical University (no. gdpulac2023001).

### Blockage of Piezo1 with GsMTx4 in the *P. berghei* ANKA-infected mice

Sixty-eight mice were randomly separated into four different groups, including Naive (*n* = 14), GsMTx4 (*n* = 14), *Pb* (*n* = 20) and *Pb* + GsMTx4 (*n* = 20) groups. In the Naive and GsMTx4 groups, each animal was i.p. injected daily with only 100 μl PBS or 1.0 mg/kg GsMTx4-a inhibitor of Piezo1 (no. HY-P1410, Med Chem Express, China) diluted in the same volume of PBS. In the *Pb* and *Pb* + GsMTx4 groups, each infected animal, which had been i.p. mono-infected with 1.0 × 10^6^ *P. berghei* ANKA-iRBCs, received daily i.p. injection of 100 μl PBS or 1.0 mg/kg GsMTx4 diluted in the same volume of PBS starting from day 1 post infection (p.i.) until the day the animal was killed. Once the *P. berghei* ANKA-infected mice exhibited signs of neurological impairment (e.g. paralysis, convulsions, coma and loss of reflex), being usually moribund at 7–11 days p.i., these animals were considered ECM mice. Body weight, survival rate and ECM incidence were monitored daily. Peripheral parasitemia was determined daily using Giemsa-stained thin blood smears from days 3 p.i. to 18 p.i. and calculated as 100% × iRBCs/total RBCs.

### Bronchoalveolar lavage fluid (BALF) and histopathology analyses

Since increased total protein of BALF is usually used as a biomarker for the onset of MA-ALI, mouse BALF was collected and total protein concentrations were determined as described previously [4]. Briefly, at each examination point (at 7 and 14 days p.i.), animals were randomly selected and killed by inhalation of isoflurane (*n *= 4–5/group). BALF was obtained by instilling and aspirating the lungs with 1.0 ml ice-cold PBS; then, it was centrifuged at 1000 g at 4 °C for 10 min. The BALF supernatant was harvested and stored at − 80 °C until usage. The total protein concentrations of BALF were ultimately measured using a bicinchoninic acid protein assay kit (Beyotime, China).

At 7 and 14 days p.i., animals (*n* = 4–5/group) were also randomly selected and killed to assess histopathological lung changes using hematoxylin and eosin (H&E) staining. The harvested lung was immediately fixed in 10% buffered formalin for 48 h, dehydrated with xylene, cleared with ethanol, embedded in paraffin and then sliced into 5-μm-thick non-sequential sections. Three non-contiguous lung slices from each animal (*n* = 4–5/group) were successively deparaffinized with xylene, rehydrated with ethanol solutions and then stained with H&E dye. More than 20 fields per lung slice were randomly selected for evaluation under a Leica DM 2500B microscope at a magnification of × 400 by two blinded pathologists. Semi-quantitative histopathological lung scores, which were determined by seven parameters (alveolar hemorrhage and congestion, alveolar edema, formation of hyaline membranes, thickening of alveolar septa, intra-alveolar inflammation, interstitial inflammation and thrombus formation), were obtained to evaluate lung inflammation and injury as described previously [24]. The score of each parameter ranged from 0 to 3 as follows: 0 = absent; 1 = mild; 2 = moderate; 3 = severe. The total score of lung histopathological change was calculated as the sum of the scores for each parameter; the highest possible score was 21.

### Determination of lung wet/dry weight ratio

Since lung wet/dry weight ratio commonly serves as an indicator of pulmonary edema, animals (*n* = 4–5/group) were randomly selected and killed to measure this ratio at 7 or 14 days p.i. Briefly, the freshly harvested right lung was immediately wiped dry using filter paper and weighed using an electronic balance. Subsequently, the lung tissue was dried in an incubator at 80 °C for 2 days and weighed again. The lung wet/dry weight ratio was calculated to evaluate the degree of pulmonary edema.

### Evaluation of lung vascular leakage

Since pulmonary vascular leakage is a hallmark of onset of MA-ALI, Evans blue staining was used to evaluate lung vascular leakage in MA-ALI mice upon GsMTx4 treatment. At 7 or 14 days p.i., 4–5 mice/group were killed by inhalation of isoflurane and then i.v. injected with 100 μl of 2% Evans blue dye for 30 min. Lungs were immediately harvested, weighted and placed in 2.0 ml formamide at 37 °C for 48 h to extract Evans blue dye from the tissue. After centrifugation at 1000 g at 4 °C for 10 min, the absorbance of Evans blue in supernatant was measured at *λ* = 620 nm using a microplate reader. Lung permeability was calculated referring to standard curves and indicated as micrograms of Evans Blue per gram of lung weight.

### TUNEL assay

TUNEL-DAPI double staining was performed to evaluate lung apoptotic changes in the experimental MA-ALI mice upon GsMTx4 treatment following the manufacturer’s instructions (no. G1501-50 T; Servicebio). Briefly, three non-contiguous lung slices from each animal (*n* = 4–5/group) were deparaffinized with xylene, rehydrated with graded ethanol solutions and subsequently incubated with 20 mg/ml of proteinase K at 37 °C for 30 min. After rinsing with distilled water, the slices were immersed in TUNEL reaction mixture (CY5-biotinylated dUTP in TdT buffer) at 37 °C for 60 min, restained with 1 μg/ml of DAPI solution at 37 °C for 10 min, coverslipped with anti-fade polyvinylpyrrolidone solution (Beyotime, China) and then examined under a fluorescence microscope. Fluorescent images of TUNEL-positive apoptotic cells with green color and cell nuclei with blue color were captured using a Leica DM 2500B microscope at × 200 magnification. The apoptosis index was determined as apoptosis-positive number/field from more than 20 random fields per lung section (*n* = 4–5 mice/group).

### Iron state in lung tissue using Perl's Prussian blue staining

To explore the effect of GsMTx4 treatment on iron state in lung tissue of the experimental MA-ALI mice, Perl's Prussian blue staining was carried out using an Iron Stain Kit based on the manufacturer’s instructions (no. G1029-100ML; Servicebio). Briefly, three non-contiguous lung slices from each animal (*n* = 4–5/group) were successively deparaffinized with xylene, rehydrated with a graded ethanol solution and subsequently cultured in a freshly prepared mixture of 5% potassium ferrocyanide and 5% hydrochloric acid for 30 min. After rinsing with double-distilled water, the slides were immersed in 2% pararosaniline solution for 5 min and sealed with neutral gum. The positively stained cells were shown by blue staining and captured using a Leica DM 2500B light microscope at × 400 magnification. The integrated optical density (IOD)/area of positive Perl's Prussian blue-stained cells was calculated in > 20 random fields per lung section (*n* = 4–5 mice/group).

### Immunohistochemistry of CD68, CD86, CD206, GPX4, 4-HNE and Piezo1 in lung

To assess the effect of GsMTx4 treatment on lung macrophage M1/M2 polarizations and ferroptosis in the experimental MA-ALI mice, immunohistochemical analysis for CD68 (a marker for all macrophages), CD86 (a marker for M1-like macrophages), CD206 (a marker for M2-like macrophages), GPX4 (an inhibitor of ferroptosis), 4-HNE (4-hydroxynonenal, an activator of ferroptosis) and Piezo1 in lung tissue across different groups was performed. Briefly, three non-contiguous lung slices from each animal (*n* = 4–5/group) were sequentially deparaffinized with xylene, rehydrated with a graded ethanol series, boiled in a microwave with sodium citrate buffer (pH 6.0) at 95 °C for 10 min for retrieval antigens and subsequently cultured with 3% hydrogen peroxide to remove endogenous peroxidase. To avoid non-specific binding, the slides were immersed in normal serum at 37 ℃ for 30 min. Following three washes with PBS, slices were sequentially incubated with primary antibodies [monoclonal rabbit anti-CD68 antibody (1:200 dilutions; no. 97778S; CST); monoclonal rabbit anti-CD86 antibody (1:200 dilutions; no. 19589S; CST); polyclonal rabbit anti-CD206 antibody (1:400 dilutions; no. GB113497-100; Servicebio); polyclonal rabbit anti-GPX4 antibody (1:100 dilutions; no. DF6701; Affinity); monoclonal mouse anti-4-HNE antibody (1:200 dilutions; no. ab48506; Abcam); or monoclonal mouse anti-Piezo1 antibody (1:200 dilutions; no. MA5-32876; Invitrogen)] followed by co-culture with secondary antibody [goat anti-rabbit IgG (H + L) HRP (1:200 dilutions; no. S0001; Affinity) or goat anti-mouse IgG HRP (1:200 dilutions; no. 8125P; CST)] at 37 ℃ for 45 min, respectively. Finally, the slides were overlaid with horseradish peroxidase (HRP)-conjugated secondary antibody (1:1000 dilution), covered with freshly prepared hematoxylin and captured using a Leica DM 2500B light microscope at × 200 and × 400 magnification. Positively stained cells were shown by dark-brown staining. The number of positively stained CD68, CD86 and CD206 cells/field in lung was calculated from > 20 random fields per lung section from each animal (*n* = 4–5/group), while IOD/area was examined to assess the level of positive expression of GPX4, 4-HNE and Piezo1.

### Immunofluorescence Double Staining for CD68^+^-Piezo1^+^ and CD68^+^-4-HNE^+^ pulmonary macrophages

To explore the effect of GsMTx4 treatment on expression of Piezo1 and 4-HNE on pulmonary CD68^+^ macrophages in experimental MA-ALI mice, double immunofluorescence staining of CD68^+^-Peizo1^+^ macrophages and CD68^+^-4-HNE^+^ macrophages was performed as described previously [25]. Briefly, after being deparaffinized with xylene and rehydrated with a graded ethanol series, three non-contiguous lung slices from each animal (*n* = 4–5/group) were immersed in 10 mM sodium citrate buffer (pH 6.0) at 95 °C for 10 min for antigen retrieval and cultured with 3% H_2_O_2_ for 5 min to eliminate endogenous peroxidase activity. After rinsing with PBS, slides were blocked with 10% normal serum at 37 °C for 30 min, incubated with monoclonal rabbit anti-CD68 antibody (1:200 dilutions; no. 97778S; CST) and monoclonal mouse anti-Piezo1 antibody (1:200 dilutions; no. MA5-32,876; Invitrogen) or monoclonal mouse anti-4-HNE antibody (1:200 dilutions; no. ab48506; Abcam) at 4 °C overnight, followed by incubation with two different kinds of secondary antibody [Alexa Fluor R488 Conjugate anti-rabbit IgG, (1:400 dilution; no. 4412S; CST) and Alexa Fluor R594 anti-mouse IgG (1:1000 dilution; no. 8890S; CST)] at 37 °C for 1 h in darkness. After washing with PBS, the slices were stained with DAPI for 5 min for dying cell nuclei, mounted using anti-fade polyvinylpyrrolidone solution (Beyotime, China) and imaged under a Leica DM 2500B light microscope at ×200 magnification. The positively stained CD68 cells appeared green, whereas positively stained Piezo1 and 4-HNE cells exhibited red fluorescence. Positively stained double CD68^+^-Piezo1^+^ and CD68^+^-4-HNE^+^ cells showed yellow fluorescence. The number of positively stained CD68^+^-Piezo1^+^ and CD68^+^-4-HNE^+^ cells per field was calculated from > 20 random lung fields for each animal (*n* = 4–5/group).

### In vitro experiment of iRBC-stimulated murine RAW264.7 macrophages subjected to GsMTx4 treatment

The murine macrophage cell line (RAW264.7) was acquired from Stem Cell Bank (Chinese Academy of Sciences, China) and cultured in DMEM medium consisting of 10% FBS (no. 26010074#, Gibco^™^) and 1% penicillin/streptomycin at 37 °C with 5% CO_2_ atmosphere. RAW264.7 cells were plated at a density of 2 × 10^5^ cells/ml in six-well plates and pretreated with Piezo1 inhibitor GsMTx4 (5 μM) for 1 h, followed by co-culture with 5.0 × 10^6^ iRBC/well for 24 h and 48 h. RAW264.7 cells treated with only PBS served as a blank group. After rinsing with ice-cold PBS buffer, the harvested pellets of RAW264.7 cells were stored in Trizol reagent (no. 9109#, TaKaRa) at − 80 °C until usage. This experiment was independently repeated five times.

### qPCR assay

Total RNA was extracted from in vitro RAW264.7 cells and in vivo lung tissues across different groups utilizing Trizol reagent (no. 9109, TaKaRa) and subsequently reverse-transcribed into cDNA using PrimeScriptTM 1st Strand cDNA Synthesis Kit (no. 6210B, TaKaRa), following the manufacturer’s protocols. The primers were designed and provided by Sangon Biotech (Shanghai, China) and are listed in Additional file 1: Table S1, including CD86, CD206, TNF-α, IL-1β, IL-4, IL-10, GPX4, 4-HNE and *P. berghei* ANKA 18S rRNA. The expression of target genes was determined using SYBR Green qPCR Master Mix (no. 6110B, TaKaRa, Japan), normalized to GAPDH and calculated by using the 2^−ΔΔCt^ method. The qPCR reaction mixture contained 5.0 µl of SYBR R Premix ExTaq TM (2 ×), 0.5 µl of each primer (10 pM), 3.0 µl of dH_2_O and 1.0 µl of preamplified cDNA (100 ng/µl). The amplification conditions were as follows: 95 °C for 5 min; 40 amplification cycles (95 °C for 5 s and 60 °C for 20 s).

### Statistical analysis

GraphPad Prism 5.0 software was used for statistical analyses, and data were expressed as mean ± SD. The differences between two groups were analyzed by independent sample t-test, whereas the differences among multiple groups were checked by one-way ANOVA test. A time series analysis test or log-rank test was carried out to compare discrepancies in body weight, parasitemia and survival time of mice accross different groups, respectively. *P* < 0.05 was taken as significant difference.

## Results

### GsMTx4 treatment prolonged host survival rate but decreased body weight loss, ECM incidence and parasite burden in the *P. berghei* ANKA-infected mice

**Fig. 1:**
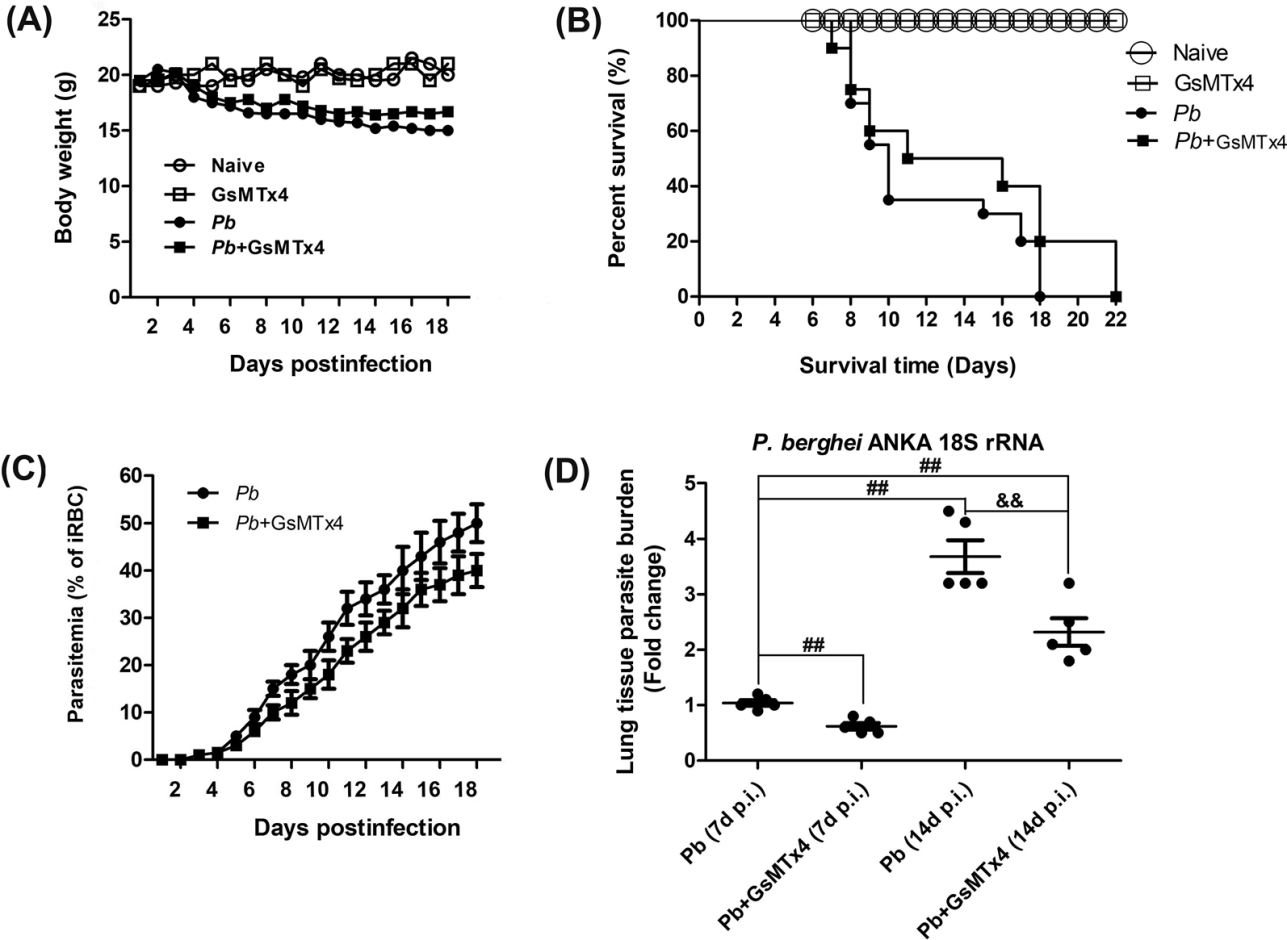
Changes in body weight, survival rate, peripheral blood parasitemia and lung parasite burden in *Plasmodium berghei* ANKA-infected mice upon GsMTx4 treatment. **A**, **B** Average body weight and survival rate were monitored daily in mice across different groups over the 18 days of observation. A time series analysis test and log-rank test was conducted to compare discrepancies in body weight or survival rate of mice, respectively. **C** Peripheral blood parasitemia was determined daily by Giemsa-stained thin blood smears in the infected mice in *Pb* and *Pb* + GsMTx4 groups. A time series analysis test was used to compare the significance of differences in parasitemia of mice. **D** Estimation of lung parasite burden at 7 and 14 days p.i. by quantifying the mRNA levels of *P. berghei* ANKA 18S rRNA using qPCR assay and 2^−ΔΔCt^ method. ^#^*P* < 0.05 and ^##^*P* < 0.01 vs. infected control mice at 7 days p.i.; ^&^*P* < 0.05 and ^&&^*P* < 0.01 vs. infected control mice at 14 days p.i. Data are expressed as mean ± SD; the experiments were performed with four to five mice per group

To explore the effect of blockage of Piezo1 with GsMTx4 on morbidity and mortality in *P. berghei* ANKA-infected mice, disease signs (e.g. body weight, ECM incidence, survival time and parasite burden) were monitored daily in mice across different groups. No significant changes or small elevations of 3–6% in body weight from the initial body weight were detected in Naive and GsMTx4 group over the 18 days of observation, whereas the infected mice in *Pb* and *Pb* + GsMTx4 groups lost body weight at 5–18 days p.i. (Fig. 1A). Notably, the infected control mice exhibited ~ 15% and ~ 22% loss relative to the initial body weight at 7 and 14 days p.i., respectively, whereas animals in the *Pb* + GsMTx4 group showed a lower body weight loss than those in the *Pb* group (~ 9% vs. ~ 15% at 7 days p.i., *P* < 0.05; ~ 16% vs. ~ 22% at 14 days p.i., *P* < 0.05). As shown in Fig. 1B, the infected control mice in *Pb* group died within 8–18 days p.i., during which ~ 65% of them developed into ECM mice. In contrast, the infected mice with GsMTx4 treatment survived longer and showed a lower ECM incidence (~ 50% vs. ~ 65%, *P* < 0.05) than those in the *Pb* group, resulting in a significant difference in survival curves of mice between *Pb* and *Pb* + GsMTx4 groups (*P* = 0.0032). As shown in Fig. 1C, a lower peripheral blood parasitemia at 5–18 days p.i. was observed in the infected mice in *Pb* + GsMTx4 group compared with those in *Pb* group (*P* < 0.05). Consistent with trends in peripheral blood parasitemia, there was a significant decline in lung parasite burden at 7 and 14 days p.i. (*P* < 0.01) in the infected mice treated with GsMTx4 compared to the infected control mice, as determined by measuring the level of *P. berghei* ANKA 18S rRNA with qRT-PCR assay (Fig. 1D). Thus, our results indicated that blockage of Piezo1 with GsMTx4 remarkably prolonged the survival rate but decreased peripheral parasitemia/lung tissue parasite burden and ECM incidence in response to *P. berghei* ANKA infection.

### GsMTx4 treatment alleviated lung damage and apoptosis in the murine model of MA-ALI

**Fig. 2:**
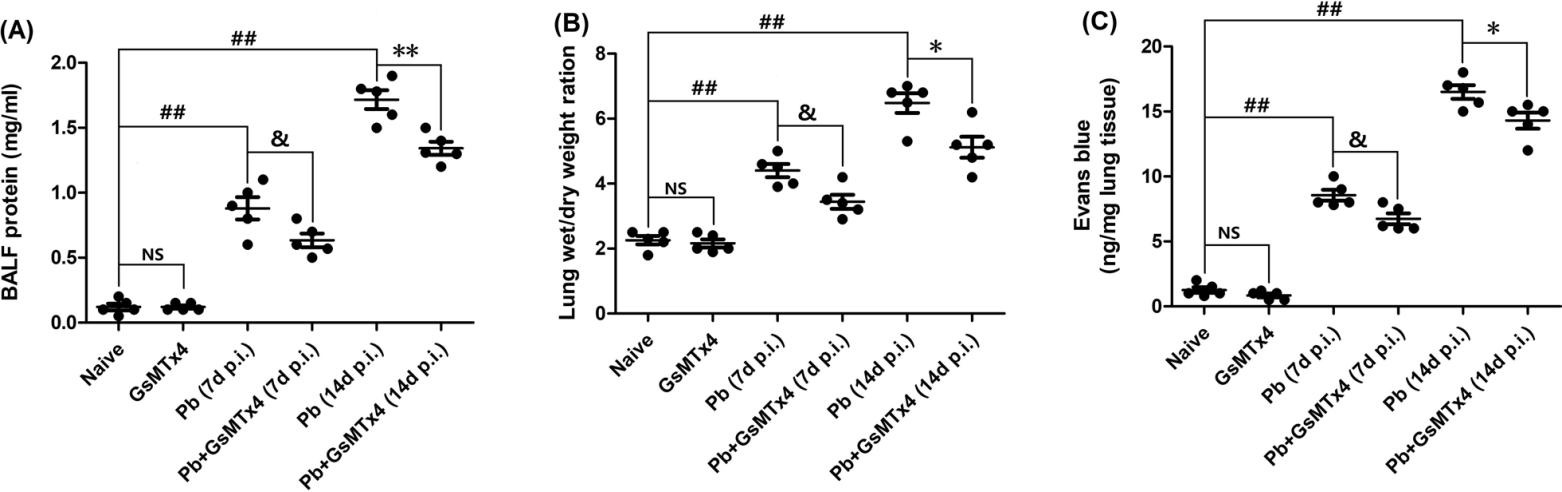
Changes in total protein in BALF, lung wet/dry weight ratio and pulmonary vascular leakage in *Plasmodium berghei* ANKA-infected mice upon GsMTx4 treatment. Four to five mice were randomly selected for analysis at 7 or 14 days p.i. **A** Total protein concentration in BALF according to BCA protein assay. **B** Lung wet weight/dry (W/D) weight ratio. **C** Pulmonary vascular leakage by Evan blue staining. The differences between two groups and among multiple groups were analyzed by independent sample t-test and one-way ANOVA test, respectively. NS, *P* > 0.05 vs. naive mice; ^#^*P* < 0.05 and ^##^*P* < 0.01 vs. naive mice; ^&^*P* < 0.05 and ^&&^*P* < 0.01 vs. infected control mice at 7 days p.i.; ^*^P < 0.05 and ^**^P < 0.01 vs. infected control mice at 14 days p.i. Data are expressed as mean ± SD; the experiments were performed with four to five mice per group

To assess the effect of GsMTx4 on the severity of MA-ALI, multiple MA-ALI determinants, including total protein concentrations in BALF, lung wet/dry weight ratio and lung vascular leakage, were analyzed across different groups. As shown in Fig. 2, GsMTx4 treatment did not notably alter these three MA-ALI determinants in the uninfected mice compared with naive mice (*P* > 0.05). However, there was a significant elevation in total protein concentrations in BALF, lung wet/dry weight ratio and lung vascular leakage in the infected control mice (*Pb* group) at 7 and 14 days p.i. compared to those in the naive group (*P* < 0.01), indicating the successful establishment of a murine model of MA-ALI in C57BL/6 mice with *P. berghei* ANKA infection. Moreover, the elevated levels of total protein concentrations in BALF (*P* < 0.05 or *P* < 0.01), lung wet/dry weight ratio (*P* < 0.05) and lung vascular leakage (*P* < 0.05) were remarkably suppressed by GsMTx4 in the infected mice at 7 or 14 days p.i. in *Pb* + GsMTx4 group when compared with *Pb* group.

**Fig. 3:**
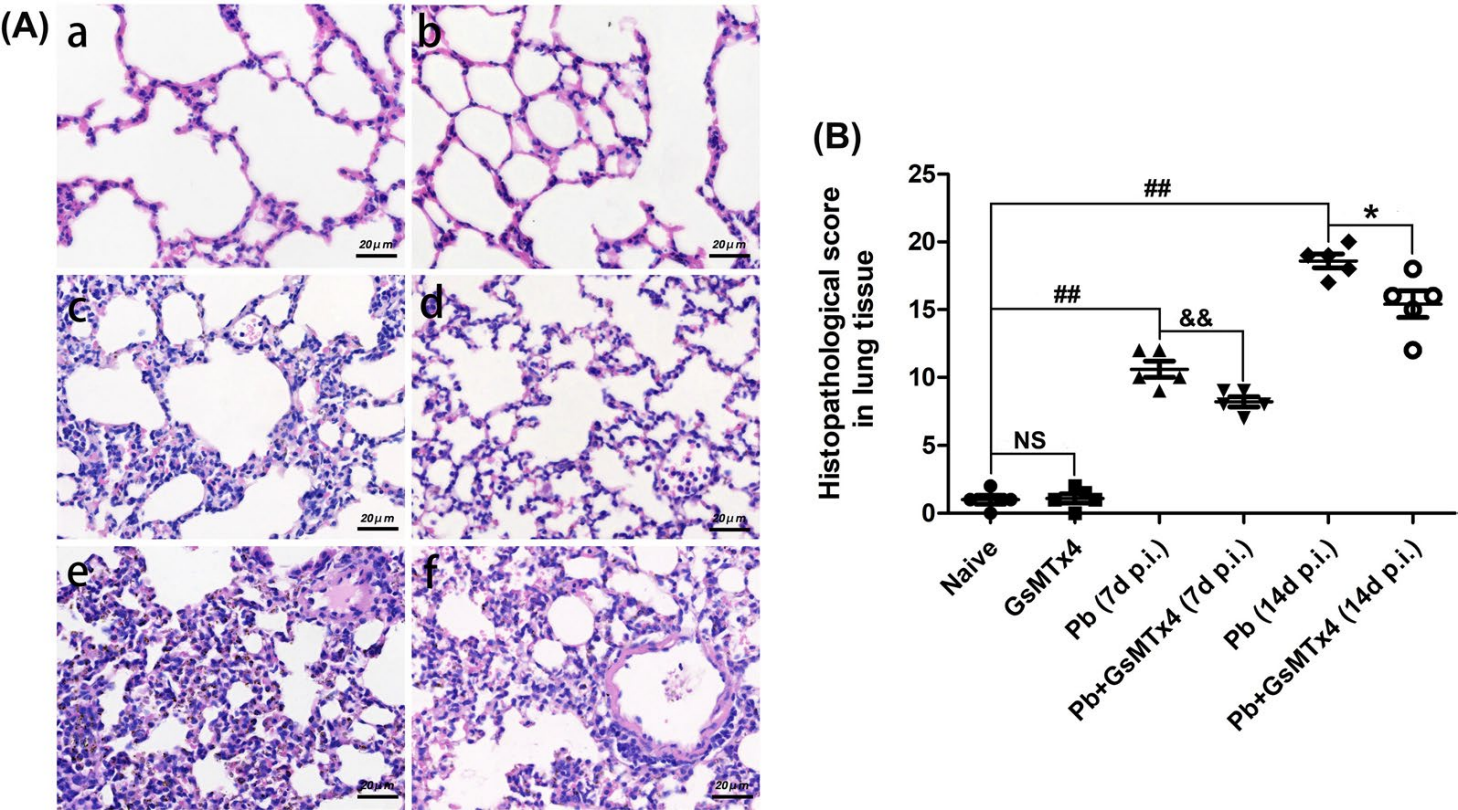
Changes in pulmonary histopathological damage in experimental MA-ALI mice upon GsMTx4 treatment. **B** Representative figures of pulmonary histopathological damage in mice across different groups using H&E staining under a light microscope at 400 × magnification. Naive mice **a**; GsMTx4-treated uninfected mice **b**; *Plasmodium berghei* ANKA-infected control mice at 7 days p.i. **c**; GsMTx4-treated infected mice at 7 days p.i. **d**; *P. berghei* ANKA infected-control mice at 14 days p.i. **e**; GsMTx4-treated infected mice at 14 days p.i. **f**. **B** Analysis of pulmonary histopathology scores. The differences in histopathology scores between two groups and among multiple groups were analyzed by independent sample t-test and one-way ANOVA test, respectively. NS, *P* > 0.05 vs. naive mice; ^#^*P* < 0.05 and ^##^*P* < 0.01 vs. naive mice; ^&^*P* < 0.05 and ^&&^*P* < 0.01 vs. infected control mice at 7 days p.i.; ^*^P < 0.05 and ^**^P < 0.01 vs. infected control mice at 14 days p.i. Data are expressed as mean ± SD; the experiments were performed with four to five mice per group

Subsequently, H&E staining was performed to identify pathological changes in the lung in the experimental MA-ALI mice following GsMTx4 treatment. As shown in Fig. 3A, no obvious morphological abnormality or pathological change was observed in lung tissue in the Naive or GsMTx4 group. Following infection at 7 or 14 days p.i., animals in *Pb* group contrarily revealed massive iRBCs in interalveolar spaces, thickened alveolar walls, obvious inflammatory cell infiltration, thrombus, pulmonary hemorrhage and edema in lung tissues, whereas these histopathological indicators were remarkably improved by GsMTx4 treatment in the infected mice in *Pb* + GsMTx4 group. In addition, a semi-quantitative score analysis further showed a significant reduction in lung histopathological scores in the GsMTx4-treated infected mice at 7 (*P* < 0.01) and 14 days p.i. (*P* < 0.05) compared to *Pb* group, respectively (Fig. 3B). Taken together, our results suggested that blockage of Piezo1 with GsMTx4 largely ameliorated pathological injury of the lung in experimental MA-ALI mice.

**Fig. 4:**
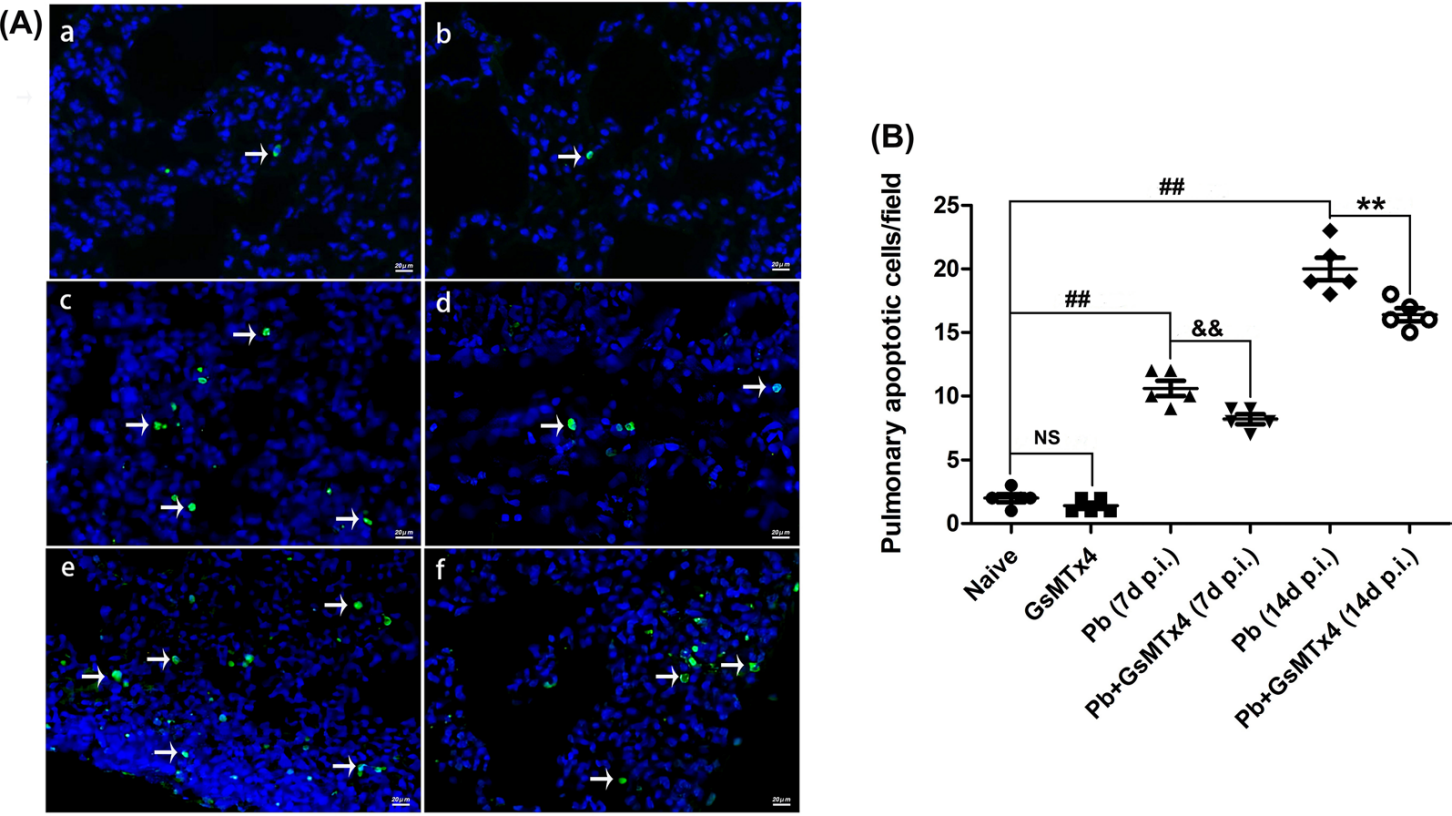
Changes in pulmonary cellular apoptosis in the experimental MA-ALI mice upon GsMTx4 treatment. **A** Representative figures of pulmonary cellular apoptosis in mice across different groups using TUNEL staining. Naive mice **a**; GsMTx4-treated uninfected mice **b**; *Plasmodium berghei* ANKA-infected control mice at 7 days p.i. **c**; GsMTx4-treated infected mice at 7 days p.i. **d**; *P. berghei* ANKA infected control mice at 14 days p.i. **e**; GsMTx4-treated infected mice at 14 days p.i. **f**. Positive apoptotic cells are illustrated by green fluorescence (arrows) and captured by fluorescence microscopy at 200 × magnification. **B** Apoptosis index is determined as positive apoptotic cells/field by counting in > 20 random lung fields per animal (*n* = 4–5/group). The differences in pulmonary apoptotic cells/field between two groups and among multiple groups were analyzed by independent sample t-test and one-way ANOVA test, respectively. NS, *P* > 0.05 vs. naive mice; ^#^*P* < 0.05 and ^##^*P* < 0.01 vs. naive mice; ^&^ *P* < 0.05 and ^&&^*P* < 0.01 vs. infected control mice at 7 days p.i.; ^*^P < 0.05 and ^**^P < 0.01 vs. infected control mice at 14 days p.i. Data are expressed as mean ± SD; the experiments were performed with four to five mice per group

Next, a TUNEL assay was performed to evaluate lung apoptosis change in the experimental MA-ALI mice upon GsMTx4 treatment. As shown in Fig. 4, rare TUNEL-positive stained cells were detected in the lung tissues in Naive and GsMTx4 groups. However, a substantial increase of pulmonary apoptotic cells/field was observed in the infected mice in *Pb* group compared to Naive group (*P* < 0.05). In contrast, a marked decline in pulmonary apoptotic cells/field was observed in *Pb* + GsMTx4 group at 7 and 14 days p.i. compared to those of *Pb* group (*P* < 0.01). These data indicated that the extensive pulmonary cellular apoptosis was significantly inhibited by GsMTx4 treatment in experimental MA-ALI mice.

### GsMTx4 treatment promoted pulmonary anti-inflammatory response in the murine model of MA-ALI

**Fig. 5:**
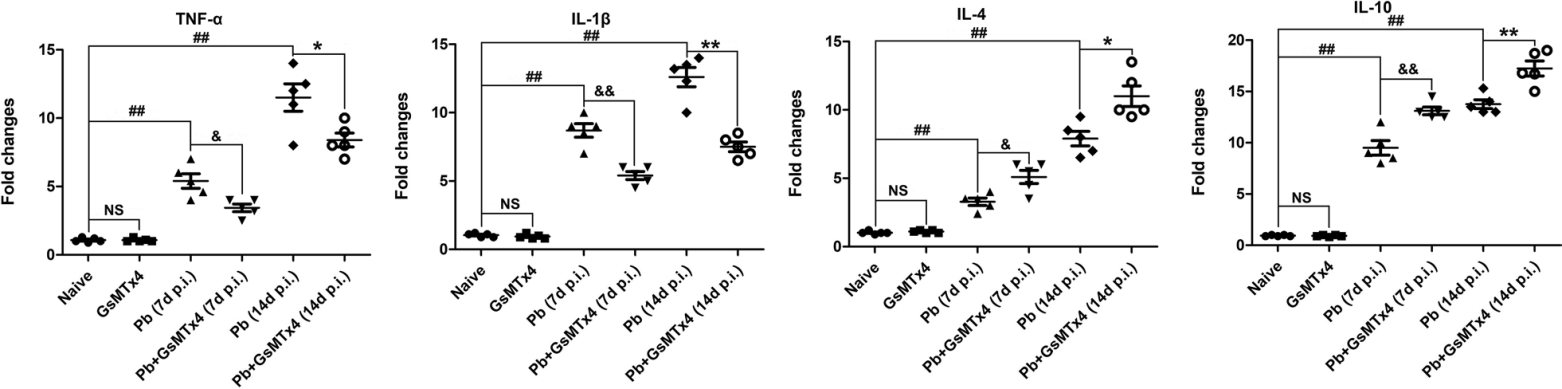
Changes in pulmonary inflammation responses in the experimental MA-ALI mice upon GsMTx4 treatment. Total RNA was collected from lung tissues in mice across different groups (*n* = 4–5/group), and the mRNA expression of target genes was quantified using qPCR assay and 2^−ΔΔCT^ method. The differences between two groups and among multiple groups were analyzed by independent sample t-test and one-way ANOVA test, respectively. NS, *P* > 0.05 vs. naive mice; ^#^*P* < 0.05 and ^##^*P* < 0.01 vs. naive mice; ^&^*P* < 0.05 and ^&&^*P* < 0.01 vs. infected control mice at 7 days p.i.; ^*^P < 0.05 and ^**^P < 0.01 vs. infected control mice at 14 days p.i. The experiment was repeated three times with similar results. Data are expressed as mean ± SD; the experiments were performed with four to five mice per group

We also assessed the changes in pulmonary inflammatory response in the experimental MA-ALI mice upon GsMTx4 treatment using a qPCR assay. As shown in Fig. 5, no significant change in the mRNA levels of all evaluated cytokines (e.g. TNF-α, IL-1β, IL-4, and IL-10) was observed in lung tissues between Naive and GsMTx4 groups (*P* > 0.05). However, increased mRNA levels of all evaluated cytokines were observed in lung tissues of *Pb*A-infected control mice at 7 and 14 days p.i. (*P* < 0.01). Notably, there was a dramatical elevation in the mRNA levels of anti-inflammatory cytokines [e.g. IL-4 (*P* < 0.05) and IL-10 (*P* < 0.01)] but a remarkable decrease of pro-inflammatory cytokines [e.g. TNF-α (*P* < 0.05) and IL-1β (*P* < 0.01)] in lung tissues in *Pb* + GsMTx4 group at 7 and 14 days p.i. compared to *Pb* group. Therefore, GsMTx4 treatment promoted a pulmonary anti-inflammatory response in the experimental MA-ALI mice.

### GsMTx4 treatment triggered pulmonary macrophage M2 polarization in the murine model of MA-ALI

**Fig. 6:**
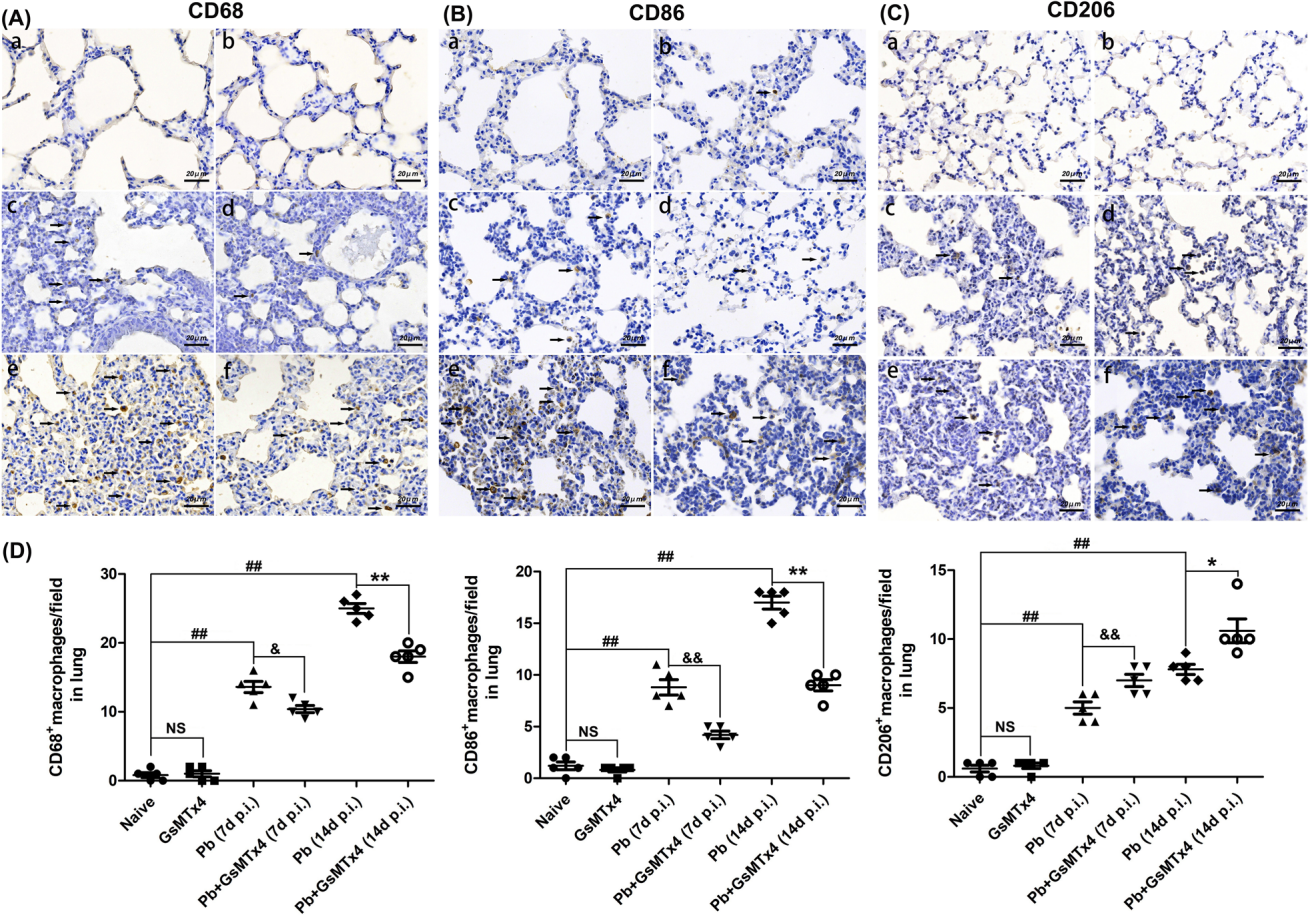
Changes in pulmonary macrophage M1/M2 polarization in the experimental MA-ALI mice upon GsMTx4 treatment. **A**–**C** Representative figures of pulmonary CD68 (a marker of total macrophages), CD86 (a marker of M1-like macrophages) and CD206 (a marker of M2-like macrophages) in mice across different groups using immunohistochemical staining under a light microscope at 400 × magnification. The positively stained CD68^+^, CD86^+^ and CD206^+^ macrophages are illustrated by dark-brown staining (arrows). Naive mice **a**; GsMTx4-treated uninfected mice **b**; *Plasmodium berghei* ANKA-infected control mice at 7 days p.i. **c**; GsMTx4-treated infected mice at 7 days p.i. **d**; *P. berghei* ANKA-infected control mice at 14 days p.i. (**e**); GsMTx4-treated infected mice at 14 days p.i. **f**. **D** Positively stained cells/field was calculated from > 20 lung fields per animal (*n* = 4–5 mice/group). The differences in positively stained cells/field between two groups and among multiple groups were analyzed by independent sample t-test and one-way ANOVA test, respectively. NS, *P* > 0.05 vs. naive mice; ^#^*P* < 0.05 and ^##^*P* < 0.01 vs. naive mice; ^&^*P* < 0.05 and ^&&^*P* < 0.01 vs. infected control mice at 7 days p.i.; ^*^P < 0.05 and ^**^P < 0.01 vs. infected control mice at 14 days p.i. Data are expressed as mean ± SD; experiments were performed with four to five mice per group

As shown in Fig. 6, immunohistochemistry staining showed that only a few positively stained pulmonary CD68^+^, CD86^+^ and CD206^+^ macrophages were found in Naive and GsMTx4 groups. During *P. berghei* ANKA infection, three types of macrophages were commonly observed in the alveolar spaces, interstitial area and even alveoli in lung tissues. The animals in *Pb* group exhibited a remarkable elevation in positively stained CD68^+^, CD86^+^ and CD206^+^ macrophages in lung tissues compared to Naive group (*P* < 0.01). However, of the macrophages, M1-like subtype was the most prevalent in lung tissues of the infected mice in *Pb* group, with frequencies of ~ 64% and ~ 68% referring to positively stained CD68^+^ macrophages at 7 and 14 days p.i., respectively. In contrast, administration of GsMTx4 dramatically decreased the numbers of CD68^+^ macrophages (*P* < 0.05 and *P* < 0.01) and CD86^+^ macrophages (*P* < 0.01) and the ration of CD86^+^ macrophages/CD68^+^ macrophages (*P* < 0.05) while significantly elevating the number of pulmonary CD206^+^ macrophages (*P* < 0.05) and ration of CD206^+^ macrophages/CD68^+^ macrophages (*P* < 0.05) in *Pb* + GsMTx4 group related to those in *Pb* group at 7 and 14 days p.i., respectively. These findings suggest that blockage of Piezo1 with GsMTx4 promoted pulmonary macrophage polarization toward M2 phenotype during the process of MA-ALI.

### GsMTx4 treatment reduced pulmonary ferroptosis in murine model of MA-ALI

**Fig. 7:**
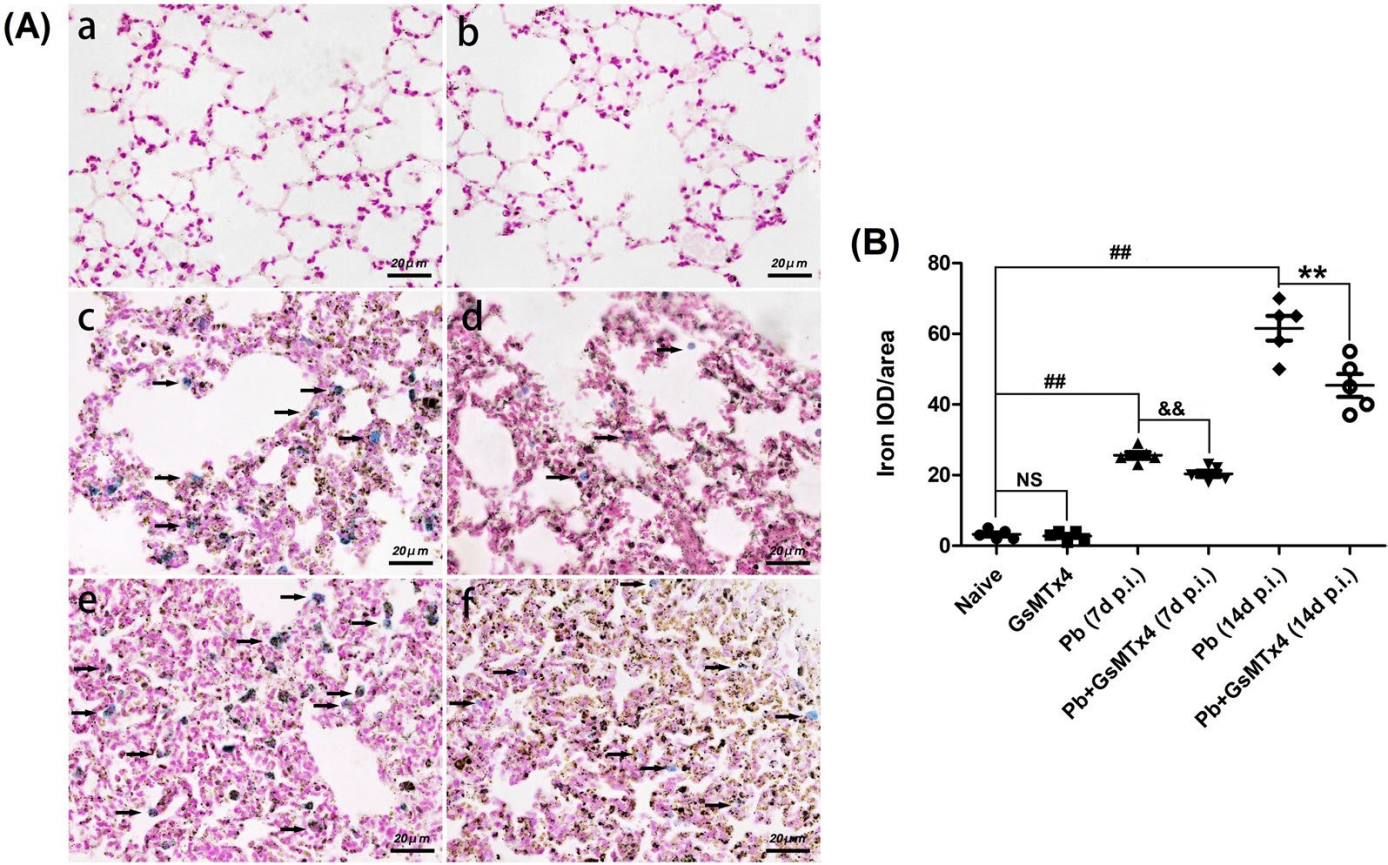
Changes in pulmonary iron accumulation in the experimental MA-ALI mice upon GsMTx4 treatment. **A** Representative figures of pulmonary iron accumulation in mice across different groups using Perl’s Prussian blue staining under light microscope at 400 × magnification. Naive mice **a**; GsMTx4-treated uninfected mice **b**; *Plasmodium berghei* ANKA-infected control mice at 7 days p.i. **c**; GsMTx4-treated infected mice at 7 days p.i. **d**; *P. berghei* ANKA-infected control mice at 14 days p.i. **e**; GsMTx4-treated infected mice at 14 days p.i. **f**. **B** IOD/area of positive Perl’s Prussian blue stained cells was calculated in > 20 lung fields per animal (*n* = 4–5 mice/group). The differences in IOD/area between two groups and among multiple groups were analyzed by independent sample t-test and one-way ANOVA test, respectively. NS, *P* > 0.05 vs. naive mice; ^#^*P* < 0.05 and ^##^*P* < 0.01 vs. naive mice; ^&^*P* < 0.05 and ^&&^*P* < 0.01 vs. infected control mice at 7 days p.i.; ^*^P < 0.05 and ^**^P < 0.01 vs. infected control mice at 14 days p.i. Data are expressed as mean ± SD; the experiments were performed with four to five mice per group

**Fig. 8:**
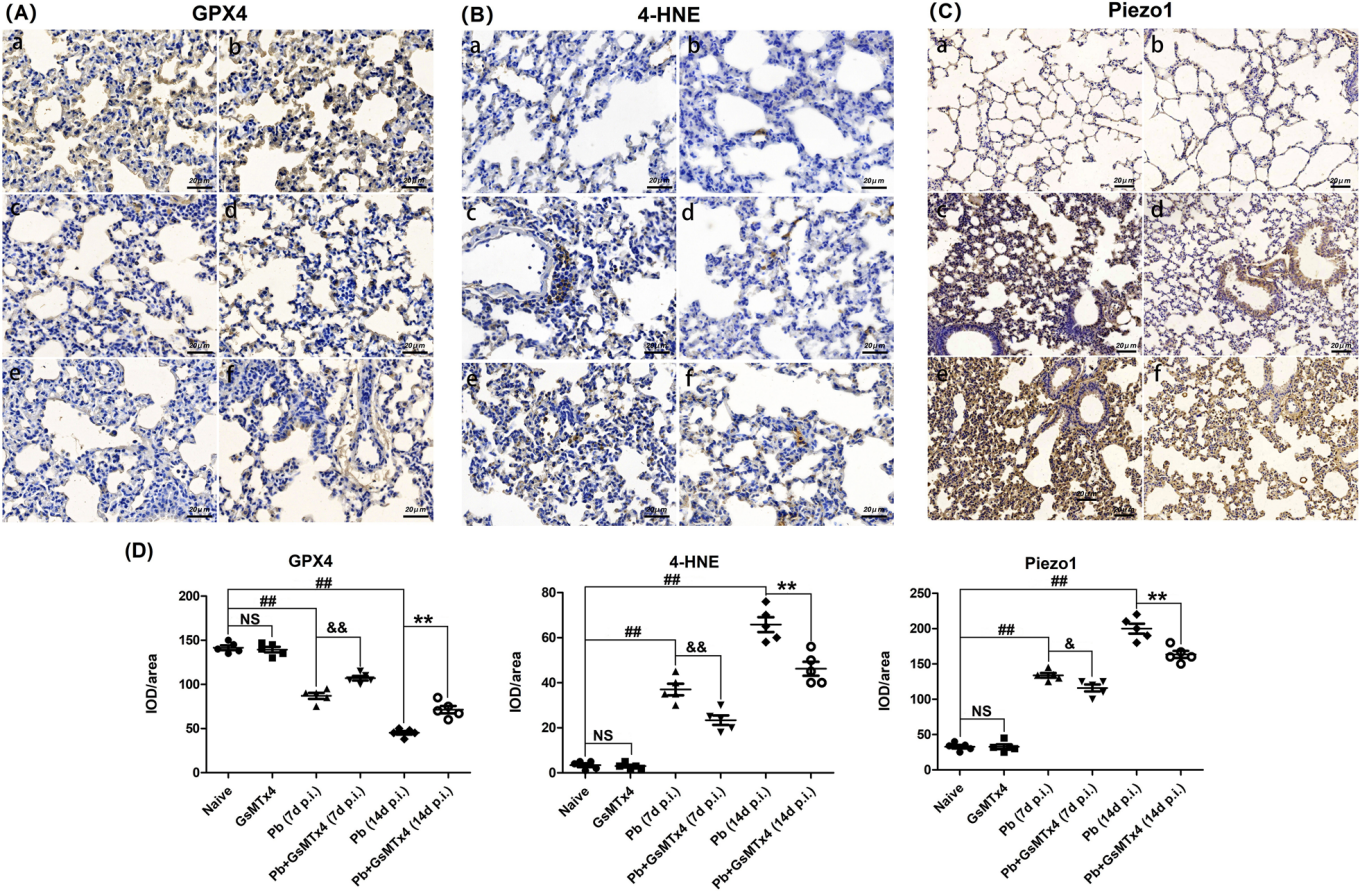
Changes in pulmonary Piezo1 and ferroptosis in the experimental MA-ALI mice upon GsMTx4 treatment. **A**–**C** Representative figures for pulmonary GPX4 (an inhibitor of ferroptosis), 4-HNE (an activator of ferroptosis) and Piezo1 in mice across different groups using immunohistochemical staining under a light microscope at 400 × and 200 × magnification. The positively stained GPX4, 4-HNE and Piezo1 cells are illustrated by dark-brown staining. Naive mice **a**; GsMTx4-treated uninfected mice **b**; *Plasmodium berghei* ANKA infected-control mice at 7 days p.i. **c**; GsMTx4-treated infected mice at 7 days p.i. **d**; *P. berghei* ANKA infected control mice at 14 days p.i. **e**; GsMTx4-treated infected mice at 14 days p.i. **f**. **D** IOD/area of positively stained GPX4, 4-HNE and Piezo1 cells was calculated in > 20 lung fields per animal (*n* = 4–5 mice/group). The differences in IOD/area between two groups and among multiple groups were analyzed by independent sample t-test and one-way ANOVA test, respectively. NS, *P* > 0.05 vs. naive mice; ^#^*P* < 0.05 and ^##^*P* < 0.01 vs. naive mice; ^&^*P* < 0.05 and ^&&^*P* < 0.01 vs. infected control mice at 7 days p.i.; ^*^P < 0.05 and ^**^P < 0.01 vs. infected control mice at 14 days p.i. Data are expressed as mean ± SD; the experiments were performed with four to five mice per group

To evaluate the effect of GsMTx4 treatment on pulmonary iron accumulation in the process of MA-ALI, Perl's Prussian blue staining was employed. As shown in Fig. 7, no obvious iron accumulation was observed in lung tissues in Naive and GsMTx4 groups, while *P. berghei* ANKA infection triggered higher levels of iron deposition in lung tissues in *Pb* group at 7 and 14 days p.i. than in Naive group (*P* < 0.01). Notably, the administration of GsMTx4 treatment dramatically reduced the level of pulmonary iron accumulation in *Pb* + GsMTx4 group compared to *Pb* group at 7 and 14 days p.i. (*P* < 0.01). Similarly, immunohistochemical staining of ferroptosis markers (e.g. GPX4 and 4-HNE) and Piezo1 was performed across different groups (Fig. 8), and the data revealed a higher expression of 4-HNE (an activator of ferroptosis, *P* < 0.01) but lower expression of GPX4 (an inhibitor of ferroptosis, *P* < 0.01) and Piezo1 (*P* < 0.05 and *P* < 0.01) in *Pb* + GsMTx4 group at 7 and 14 days p.i. compared to *Pb* group. Our results demonstrated that blockage of Piezo1 with GsMTx4 suppressed pulmonary ferroptosis during the MA-ALI process.

### GsMTx4 treatment decreased Piezo1 and 4-HNE expression in CD68^+^ macrophages in murine model of MA-ALI

**Fig. 9:**
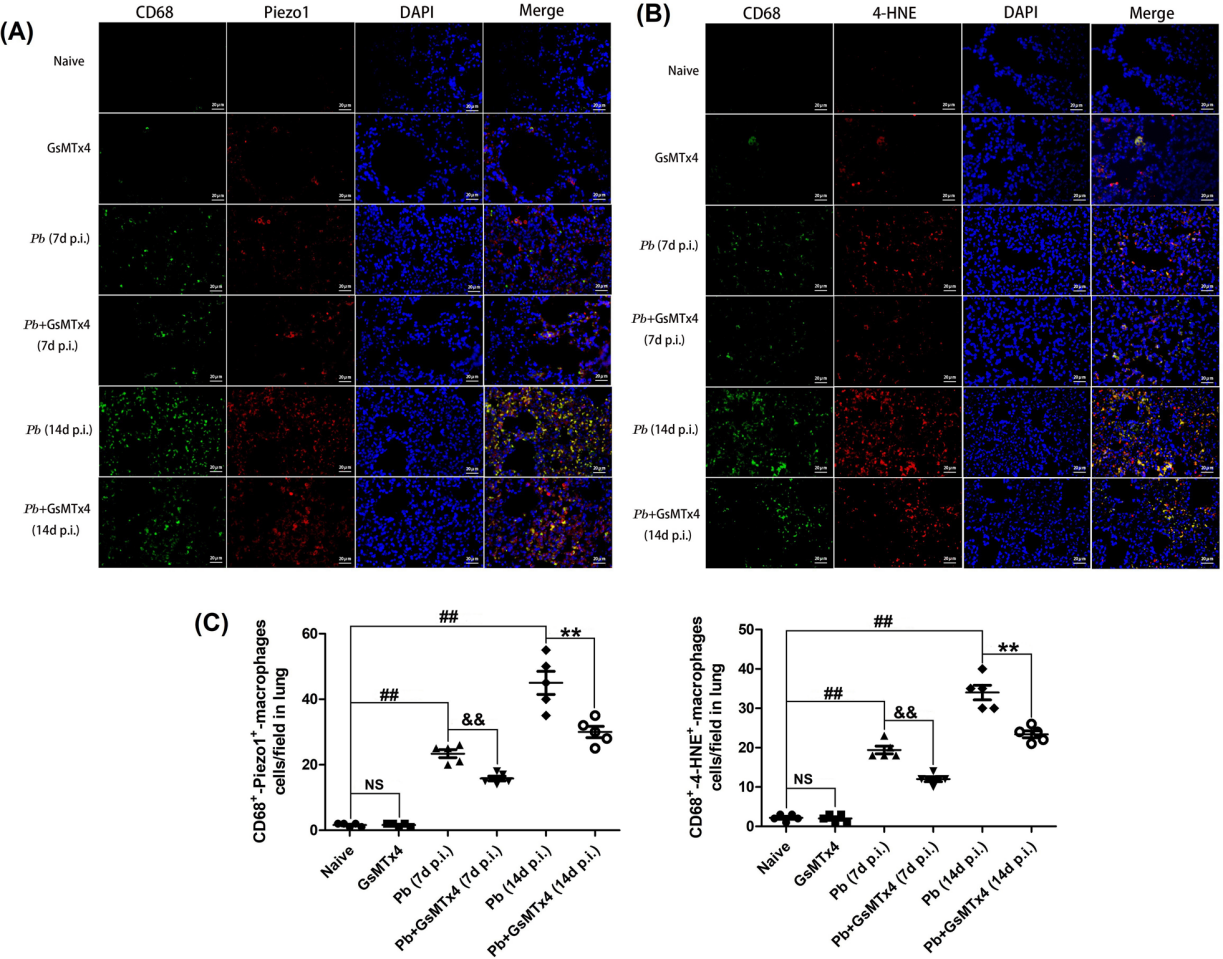
Changes in pulmonary CD68^+^-Piezo1^+^ and CD68^+^-4-HNE^+^ macrophage number in the experimental MA-ALI mice upon GsMTx4 treatment. **A** Representative figures of pulmonary Piezo1 and 4-HNE expression in CD68^+^ macrophages in mice across different groups using immunofluorescence double staining under a fluorescence microscope at 200 × magnification. Positive expression of CD68^+^ macrophages is shown by green fluorescence and positive expression of Piezo1 and 4-HNE by red fluorescence. Co-expression of CD68^+^ macrophage and Piezo1 (or 4-HNE) was indicated by yellow fluorescence. **B** Number of CD68^+^-Piezo1^+^ macrophages/field and CD68^+^-4-HNE^+^ macrophages/field was counted from > 20 lung fields per animal (*n *= 4–5 mice/group). Differences in numbers of CD68^+^-Piezo1^+^ macrophages/field and CD68^+^-4-HNE^+^ macrophages/field between two groups and among multiple groups were analyzed by independent sample t-test and one-way ANOVA test, respectively. NS, *P* > 0.05 vs. naive mice; ^#^*P* < 0.05 and ^##^*P* < 0.01 vs. naive mice; ^&^*P* < 0.05 and ^&&^*P* < 0.01 vs. infected control mice at 7 days p.i.; ^*^P < 0.05 and ^**^P < 0.01 vs. infected control mice at 14 days p.i. Data are expressed as mean ± SD; the experiments were performed with four to five mice per group

We studied the Piezo1 and 4-HNE expression on pulmonary CD68^+^ macrophages in the experimental MA-ALI mice upon GsMTx4 treatment. Immunofluorescence double staining showed that a few pulmonary CD68^+^-Piezo1^+^ macrophages and CD68^+^-4-HNE^+^ macrophages were found in Naive and GsMTx4 groups (Fig. 9). *Pb*A-infected control mice exhibited a significant elevation in the number of pulmonary CD68^+^-Piezo1^+^ (*P* < 0.01) and CD68^+^-4-HNE^+^ macrophages (*P* < 0.01) at 7 and 14 days p.i. relative to naive mice, respectively. Notably, the number of pulmonary CD68^+^-Piezo1^+^ macrophages/field and CD68^+^-4-HNE^+^ macrophages/field was reduced by GsMTx4 treatment in *Pb* + GsMTx4 groups compared with *Pb* group (*P* < 0.01). Collectively, these results indicated that blockage of Piezo1 with GsMTx4 inhibited Piezo1 and ferroptosis expression in pulmonary CD68^+^ macrophages during the MA-ALI process.

### GsMTx4 treatment led to higher mRNA levels of M2 polarization markers and GPX4 but a lower level of 4-HNE in the iRBC-stimulatd RAW264.7 cells in vitro

**Fig. 10:**
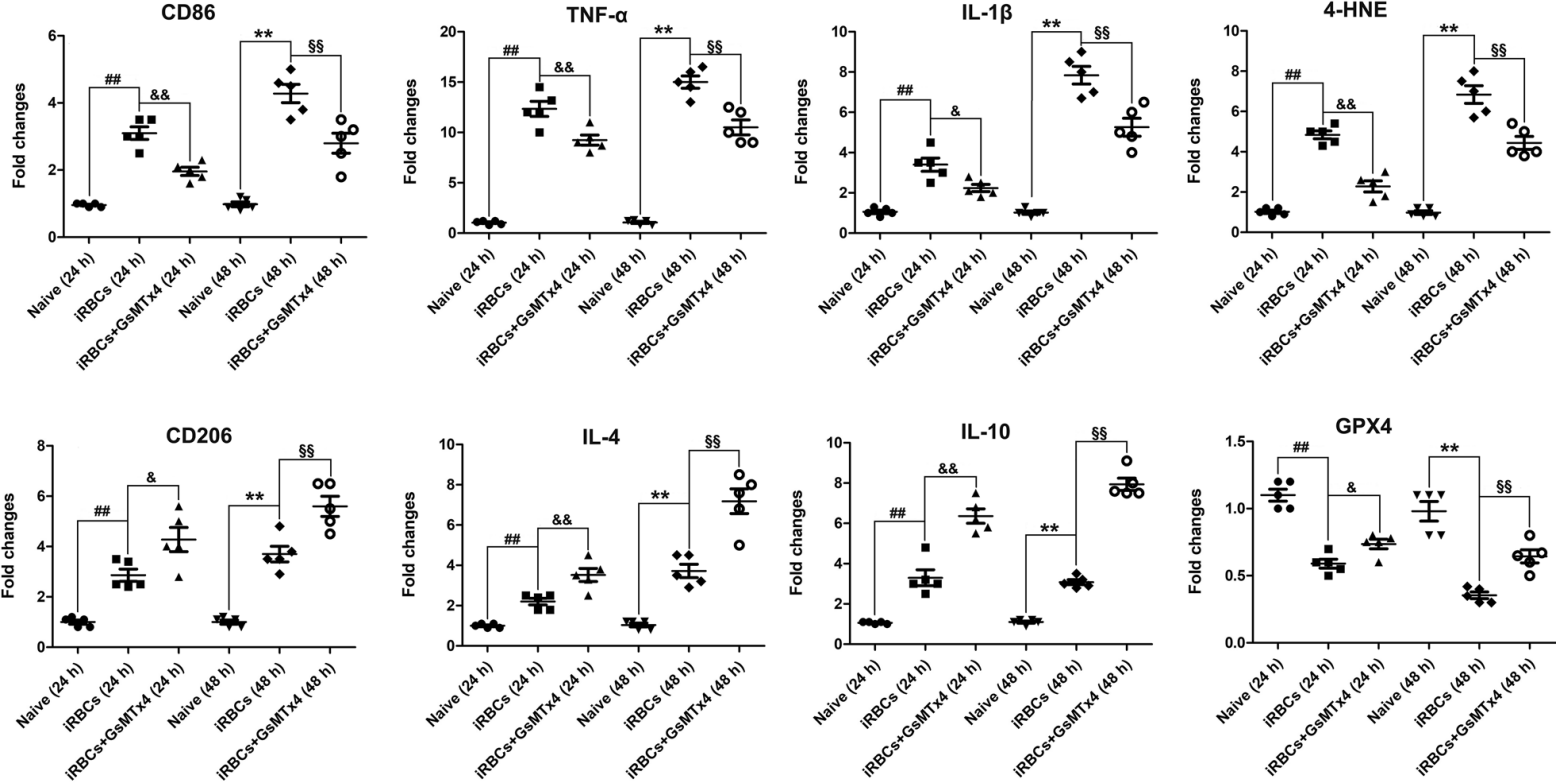
Changes in mRNA expression in CD86, CD206, TNF-α, IL-1β, IL-4, IL-10, GPX4 and 4-HNE in iRBC-stimulated RAW264.7 cells upon GsMTx4 treatment. RAW264.7 cells were pretreated with Piezo1 inhibitor GsMTx4 (5 μM) for 1 h followed by co-culture with 5.0 × 10^6^ iRBC/well for 24 h and 48 h, respectively. Total RNA was collected from RAW264.7 cells across different groups, and the mRNA expression of target genes was quantified by using qPCR assay and 2^−ΔΔCT^ method. The differences between two groups and among multiple groups are analyzed by independent sample t-test and one-way ANOVA test, respectively. ^#^*P* < 0.05 and ^##^*P* < 0.01 vs. RAW264.7 cells co-cultured only with PBS for 24 h; ^&^*P* < 0.05 and ^&&^*P* < 0.01 vs. RAW264.7 cell co-cultured with iRBCs for 24 h; ^*^P < 0.05 and ^**^P < 0.01 vs. RAW264.7 cell co-cultured only with PBS for 48 h; ^§^*P* < 0.05 and ^§§^*P* < 0.01 vs. RAW264.7 cell co-cultured with iRBCs for 48 h. Data are expressed as mean ± SD; the experiment was repeated five times with similar results

To explore the effects of GsMTx4 treatment on polarization and ferroptosis in iRBC-stimulated RAW264.7 cells in vitro, the mRNA levels of CD86, CD206, TNF-α, IL-1β, IL-4, IL-10, GPX-4 and 4-HNE in iRBC-stimulated RAW264.7 cells upon GsMTx4 treatment were measured using qPCR assay (Fig. 10). Compared with RAW264.7 cells in the blank group, mRNA levels of CD86 (*P* < 0.01), CD206 (*P* < 0.01), TNF-α (*P* < 0.01), IL-1β (*P* < 0.01), IL-4 (*P* < 0.01), IL-10 and 4-HNE (*P* < 0.05) were remarkably elevated but showed notably decreased mRNA levels of GPX4 (*P* < 0.01) in the iRBC-stimulated RAW264.7 cells at 24 and 48 h of co-culture. Notably, higher mRNA levels of CD206 (*P* < 0.05 or *P* < 0.01), IL-4 (*P* < 0.01), IL-10 (*P* < 0.01) and GPX4 (*P* < 0.05 or *P* < 0.001) but lower mRNA levels of CD86 (*P* < 0.01), TNF-α (*P* < 0.01), IL-1β (*P* < 0.05 or *P* < 0.01) and 4-HNE (*P* < 0.01) were observed in the iRBC-stimulated RAW264.7 cells with GsMTx4 treatment than in those with iRBC-stimulated RAW264.7 cells at 24 and 48 h of co-culture, respectively.

## Discussion

As reported, sequestrations of iRBCs on lung postcapillary venules were documented as a pivotal factor in the pathological process of MA-ALI by partly triggering impaired blood flow and subsequent excessive pro-inflammatory responses [7, 26]. Accumulated studies have shown that the mechanosensitive Piezo1 channel was highly distributed in the organs (e.g. lung) and numerous immune cells (e.g. macrophages) and recently served as a key determinant of vascular blood flow sensitivity and subsequent lung pathology [20, 22]. A previous study further indicated that perivascular macrophages precisely regulated blood flow through the release of iNOS-produced NO following ischemic injury [22]. Thus, it is reasonably hypothesized that Piezo1 in macrophages could potentially mediate the pathogenesis of MA-ALI by sensing impaired pulmonary blood flow, but its functional role and underlying mechanism were less well understood. In the present study, our findings showed that *P. berghei* ANKA-infected mice exhibited higher levels of protein concentrations in BALF, lung wet/dry weight ratio, pulmonary vascular leakage and pathological score in lung tissue than navie mice, indicating successful establishment of the MA-ALI murine model. Our data were similar to those in a previous report [24] where *P. berghei* ANKA induced ALI in female Kunming mice; the model was successfully constructed with characterization by dyspnea or respiratory insufficiency occurring at 5–7 days p.i. before death. Compared with naive mice, elevated expression of Piezo1 and number of CD68^+^-Piezo1^+^ macrophages was observed in the lung tissue of experimental MA-ALI mice, followed by a rapid increase in lung pathological injury in the present study, indicating that the activity of Piezo1 could mediate the severity of murine MA-AIL mice. However, not all results were well understood. Previous studies demonstrated that upregulation of Piezo1 was associated with an increase in lung vascular hyperpermeability, pulmonary hypertension and ARDS-associated pulmonary fibrosis [27–29]. To further assess whether blockage of Piezo1 could improve the severity in experimental MA-ALI mice, these animals were treated with Piezo1 inhibitor GsMTx4 in the present study. Strikingly, treatment with GsMTx4 dramatically alleviated lung pathological damage and apoptosis, coinciding with a longer survival time and higher expression of anti-inflammatory cytokines (e.g. IL-4 and IL-10) but lower expression of pro-inflammatory cytokines (e.g. TNF-a and IL-1β) compared with the infected control mice. Our data were consistent with those in the previous report [11] where mice lacking Piezo1 exhibited a decreased pulmonary inflammation response in the context of fibrotic autoinflammation or bacterial infection. Additionally, pharmacological inhibition of Piezo1 with GsMTx4 alleviated lung pathological changes, water content and protein leakage in the process of MV-exacerbated ARDS-associated pulmonary fibrosis [30]. Further studies identified Piezo1 as a mediator of macrophage proliferation and polarization and demonstrated that activity of the Piezo1 channel was strongly involved in the process of macrophage-mediated inflammatory diseases [21, 31]. The data in the present study also showed that blockage of Piezo1 with GsMTx4 dramatically decreased the number of CD68^+^ and CD86^+^ macrophages (M1-like macrophages) in lung tissue but triggered a remarkable increase of CD206^+^ macrophages (M2-like macrophages) in the process of MA-ALI. Additionally, an in vitro study showed that administration of GsMTx4 led to a dramatic increase in the mRNA levels of CD206 and anti-inflammatory cytokines (e.g. IL-4 and IL-10) in iRBC-stimulated RAW264.7 cells but triggered lower mRNA levels of CD68 and pro-inflammatory cytokines (e.g. TNF-α and IL-1β). Our data were consistent with a previous report where the activated Piezo1 contrarily triggered M1 macrophage polarization in response to LPS, triggered overproduction of proinflammatory cytokines (e.g. TNF-α and IL-1β) and ultimately contributed to collagen degradation in periodontitis [32]. It was reported that the excessive pulmonary pro-inflammatory responses were strongly correlated with the pathogenesis of MA-ALI [24], while contrarily anti-inflammatory cytokines (e.g. IL-4 and IL-10) effectively improved uncontrolled pro-inflammatory response-driven lung pathogenesis [33]. Thus, our data indicated that blockage of Piezo1 with GsMTx4 could attenuate the severity of MA-ALI by promoting the pulmonary macrophages' polarization of M1 to M2 phenotype and triggering an elevated level of anti-inflammatory responses for lung tissue repair.

Apoptosis is a well-recognized form of programmed cell death that plays a pivotal role in maintaining cellular homeostasis by eliminating damaged or dysfunctional cells. Contrarily, dysfunction or dysregulation of cell apoptosis led to pathological conditions in numerous lung diseases [34]. The accumulated evidence showed that MA-ALI was partly characterized by excessive and uncontrolled inflammation and apoptosis in lung tissue [35, 36]. A substantial increase in apoptosis or apoptotic biomarkers (e.g. TNF, FAS, Bax, Bad, FAS/FASL and caspases-3/8) was commonly observed in leukocytes, alveolar cells and endothelial cells of lung tissue from severe *P. falciparum* malaria patients with pulmonary edema and experimental MA-ALI mice [35, 36]. An in vitro study also showed that apoptosis was detected in human primary pulmonary endothelial cells co-cultured with *P. falciparum* field isolates [37]. Further study demonstrated that apoptosis in endothelial cells contributed to the pathogenesis of MA-ALI, primarily as a facilitator of the alveolar-capillary barrier disruption [36]. The present study found that a dramatic elevation of apoptotic cells was observed in lung tissue of experimental MA-ALI mice compared to naive mice, while increased levels of apoptotic cells were suppressed by GsMTx4 treatment. A previous study indicated that Piezo1 mediated Ca^2+^-dependent cell death, including apoptosis and ferroptosis, in response to mechanical stimuli [38]. Our data were like those in other reports where activation of Piezo1 contrarily triggered intensive apoptosis of type II pneumocytes during ARDS [39]. Thus, our findings suggested that intervention of Piezo1 with GsMTx4 could attenuate lung injury in experimental MA-ALI mice via suppression of excessive pulmonary apoptosis. As reported above, intervention of Piezo1 with GsMTx4 dramatically promoted pulmonary macrophage polarization of M1 to M2, followed by a decrease of apoptosis and inflammatory responses in lung tissue of experimental MA-ALI mice in the present study. Similarly, it was reported that M1-like macrophages triggered an elevated level of apoptosis in bone marrow mesenchymal stem cells by delivering exosomal miR-222 to BMSCs [40], while M2-like macrophages contrarily suppressed the levels of apoptosis and inflammation in human umbilical vein endothelial cells by exosomal miR-221-3p overexpression [41]. Thus, our data indicated that intervention with Piezo1 with GsMTx4 could attenuate inflammatory responses and apoptosis in a murine model of MA-ALI by promoting macrophages M2 polarization.

Iron is an essential metal for several vital cellular processes, including DNA synthesis, cellular respiration, cell growth and death, while iron overload in organs is linked to a variety of lung diseases [42]. The previous study demonstrated that iron could prevent the ECM process by attenuating the accumulation of T cells in the brain [43]; however, another study indicated that iron overload in trophoblasts of *P. berghei*-infected placenta was related to fetal death [44], indicating a controversial role of iron overload in regulating malaria disease severity. Ferroptosis is a newly emerging type of cell death that is mainly triggered by excessive intracellular iron accumulation and massive lipid peroxidation, downregulation of system Xc− activity, inhibition of glutathione peroxidase 4 (GPX4) and an increase of lipid ROS and 4-HNE [45]. Emerging evidence has demonstrated that defective or excessive ferroptosis is involved in the pathological process of lung diseases [46]. A previous study revealed that ferroptosis contributed to neuron damage in ECM pathogenesis and activated CD8^+^T cells acted as key inducers of neuronal ferroptosis [47]. Notably, high levels of HO-1 (an activator of ferroptosis), which could induce iron overload and cell death in the context of certain inflammatory conditions [48], were detected in severe malaria patients with ALI [49]. In the present study, a dramatic increase in levels of intracellular iron accumulation and 4-HNE (an activator of ferroptosis) but decreased levels of GPX4 (an inhibitor of ferroptosis) were observed in lung tissue of experimental MA-ALI mice compared to naive mice, which were rescued by administration of the Piezo1 inhibitor GsMTx4, indicating that ferroptosis could be involved in mediating the severity of MA-ALI. Similarly, Piezo1 channel mediated ionizing radiation-induced pulmonary endothelial cell ferroptosis [50]. Previous studies demonstrated that Piezo1 was robustly expressed in macrophages and functioned as a key regulator of macrophage phagocyte activity, iron overload and subsequent cell ferroptosis [51]. Our data also demonstrated that the level of ferroptosis in CD68^+^ macrophages (or the number of CD68^+^-4-HNE^+^ macrophages/field) was decreased by administration of the Piezo1 inhibitor (GsMTx4), followed by a decline in the number of M1-like macrophages and improved lung injury in experimental MA-ALI mice. Our finding was similar to those of other previous reports, where uridine alleviated sepsis-induced acute lung injury by inhibiting ferroptosis of macrophages [52]; activated ferroptosis in macrophages contrarily accelerated the occurrence of sepsis [53]. Moreover, a previous study demonstrated that ferroptosis was triggered by increasing M1-like macrophages in neutrophilic airway inflammation, whereas quercetin decreased the level of ferroptosis in lung tissues by suppressing the pro-inflammatory M1-like macrophages [54]. Thus, our raw data suggested that blockage of Piezo1 with GsMTx4 could suppress macrophage ferroptosis and M1 polarization, in turn improving the severity of MA-ALI.

This study has several limitations: (i) Our limited facilities did not allow us to directly determine the change in pulmonary blood flow during *P. berghei* ANKA infection and identify the link between the activity of Piezo1 and change in pulmonary blood flow; (ii) Piezo1-depleted or KO mice were not suited to exploring the function of Piezo1 in the severity of MA-ALI; (iii) the present study did not accurately elucidate how Piezo1 exclusively targeted macrophage ferroptosis or M1/M2 polarization in the process of MA-ALI. Thus, further in-depth investigations without these limitations could be useful to better understand the role of Piezo1 or Piezo1 macrophages in the pathogenesis of MA-ALI.

## Conclusions

In summary, we identified the function of mechanosensitive Piezo1 channel in mediating the severity of experimental MA-ALI. Results from the present study showed a dramatically increased expression of Piezo1 and number of CD68^+^-Piezo1^+^ macrophages in lung tissues of the experimental MA-ALI mice followed by a substantial increase in lung pathological injury. The data further showed the blockage of Piezo1 with GsMTx4 alleviated lung injury in experimental MA-ALI mice, partly by promoting macrophage polarization of M1 to M2 subtypes and subsequent anti-inflammatory responses, but alternatively inhibited apoptosis and ferroptosis in lung tissues. Although the exclusive mechanism underlying Piezo1 mediates macrophage functions (e.g. M1/M2 polarization, apoptosis and ferroptosis) and its potential role in severity of MA-ALI requires further investigation, our findings suggest that inhibition of Piezo1 channel in macrophages could be a potential therapeutic target for treating MA-ALI.

### Supplementary Information

**Additional file 1: Table S1.** Sequences of target gene primers for qPCR assay.

## Data Availability

All datasets generated for this study are included in the manuscript and additional file.
