# Supplementary material for: Blockage of mechanosensitive Piezo1 channel alleviates the severity of experimental malaria-associated acute lung injury
Source: Parasit Vectors. 2024 Feb 1;17:46. doi: 10.1186/s13071-024-06144-5 (PMC10832208; doi:10.1186/s13071-024-06144-5)
Supplement: Supplementary file 1 — Additional file 1: Table S1. Sequences of target gene primers for qPCR assay. [file 13071_2024_6144_MOESM1_ESM.docx]

| Genes | Primers ^a^ |
| --- | --- |
| CD68 | F: 5’-CTCTTGCTGCCTCTCATCATTGG-3’  R: 5’-GCTGGTAGGTTGATTGTCGTCTG-3’ |
| CD86 | F: 5’-AGCACTATTTGGGCACAGAGAAAC-3’  R: 5’-GTGAAGTCGTAGAGTCCAGTTGTTC-3’ |
| CD206 | F: 5’- TCTGGTGAACGGAATGATTGTGTAG-3’  R: 5’- GCTTTGGTTGTAATGGATGAGTGTG-3’ |
| TNF-α | F: 5’- CACGCTCTTCTGTCTACTGAACTTC-3’  R: 5’- CTTGGTGGTTTGTGAGTGTGAGG-3’ |
| IL-1β | F: 5’- TCGCAGCAGCACATCAACAAG-3’  R: 5’- TCCACGGGAAAGACACAGGTAG-3’ |
| IL-4 | F: 5’- TTTGAACGAGGTCACAGGAGAAGG-3’  R: 5’- GCACCTTGGAAGCCCTACAGAC-3’ |
| IL-10 | F: 5’- GGTTGCCAAGCCTTATCGGAAATG-3’  R: 5’- GCCGCATCCTGAGGGTCTTC-3’ |
| GPX4 | F: 5’- CCCGATATGCTGAGTGTGGTTTAC-3’  R: 5’- TTTCTTGATTACTTCCTGGCTCCTG-3’ |
| 4-HNE | F: 5'-GAAGACCTCTCAGGATGACATGG-3'  R: 5'-GGTAGTGGTGGGATGTTGAG-3' |
| 1. *berghei* ANKA   18S rRNA | F: 5’- ACGGGGAGCAAGAGCAGTATTTC-3’  R: 5’- CCCCACCAAAACCTGCCTTTATTG-3’ |
| GAPDH | F: 5’- ACGGCAAATTCAACGGCACAG-3’  R: 5’- ACACCAGTAGACTCCACGACATAC-3’ |

**Additional file 1: Table 1. Sequences of target genes primers for qPCR assay.**

Notes: ^a^ F, Forward primer; R, Reverse primer.
